# Letters to the Editor

**Published:** 1987-11

**Authors:** D. W. Barritt, J. P. Mitchell

**Affiliations:** Abbey Cottage, Parry's Close, Bristol BS9 1AW; Abbey Cottage, Parry's Close, Bristol BS9 1AW


*Sir,*
*Mobility Allowance*
As consultant members of Medical Appeal Tribunals we are with increasing frequency required to agree or reject claims for mobility allowance. A proportion of these claims are difficult to decide, but in many cases the claimant or his medical advisors are unclear as to the regulations, which must be satisfied before mobility allowance can be granted. Rejection of the claim leads to disappointment or grievous dissatisfaction, particularly when the claimant has been told by "an expert" that he or she ought to have the mobility allowance.

Basically one of two requirements must be satisfied. The first is inability to walk (even with aids) or virtual inability to walk. Those quite unable to walk rarely need to bring their case to appeal. The commonest reason for dispute is when the claimant can walk short distances, but with greater or lesser difficulty or discomfort. The regulations state that "Any walking that can be achieved only with great pain or discomfort shall be discounted". Clearly the assessment of the degree of pain or discomfort is a subjective judgement and may be contentious but the law implies that the discomfort must be so severe as to amount to inability to walk at all.

Those who are able to walk can qualify for the allowance if the exertion required to walk "constitutes a danger to the patient's life or would be liable to lead to a serious deterioriation in health". In our experience this situation is uncommon as the medical advice in conditions such as severe arthritis or advanced heart disease is usually to keep mobile rather than making no effort to move.

Personal hardship in terms of finance or inaccessability of the place of residence must be disregarded.

Our purpose in writing this letter is not to discourage our colleagues from helping those who are likely to satisfy the regulations, but to restate the restrictive terms of the law. Sometimes doctors, who, are expert in their speciality write supportive letters for their patients to submit in pursuit of their claim. However sympathetic we are with the claimant's hardship only facts which relate to the degree of discomfort or difficulty in walking or the likelihood that the effort of walking may endanger health are relevant.

We see many sad and even distressing appeals for mobility allowance, but there are no discretionary powers beyond the regulations which are legally binding.

D. W. Barritt
J. P. Mitchell
Abbey Cottage,
Parry's Close,
Bristol BS9 1AW.

